# MicroRNA-Offset RNA Alters Gene Expression and Cell Proliferation

**DOI:** 10.1371/journal.pone.0156772

**Published:** 2016-06-08

**Authors:** Jin Zhao, Gavin R. Schnitzler, Lakshmanan K. Iyer, Mark J. Aronovitz, Wendy E. Baur, Richard H. Karas

**Affiliations:** Molecular Cardiology Research Institute, Tufts Medical Center, Boston, MA, 02111, United States of America; University of Massachusetts Medical, UNITED STATES

## Abstract

MicroRNA-offset RNAs (moRs) were first identified in simple chordates and subsequently in mouse and human cells by deep sequencing of short RNAs. MoRs are derived from sequences located immediately adjacent to microRNAs (miRs) in the primary miR (pri-miR). Currently moRs are considered to be simply a by-product of miR biosynthesis that lack biological activity. Here we show for the first time that a moR is biologically active. We demonstrate that endogenous or over-expressed moR-21 significantly alters gene expression and inhibits the proliferation of vascular smooth muscle cells (VSMC). In addition, we find that miR-21 and moR-21 may regulate different genes in a given pathway and can oppose each other in regulating certain genes. We report that there is a “seed region” of moR-21 as well as a “seed match region” in the target gene 3’UTR that are indispensable for moR-21-mediated gene down-regulation. We further demonstrate that moR-21-mediated gene repression is Argonaute 2 (Ago2) dependent. Taken together, these findings provide the first evidence that microRNA offset RNA alters gene expression and is biologically active.

## Introduction

MicroRNA offset RNAs (moRs) were first reported in 2009 in a simple chordate ascidian *Ciona intestinalis* as approximately 20-nt-long RNAs that are derived from sequences located immediately adjacent to microRNAs (miRs) in the primary miRs (pri-miRs) by sequencing small RNA [1]. The discovery of moRs was unexpected since originally the investigators sought to identify novel miRs from the simple chordate through comprehensive sequencing. Subsequently, moRs were identified in mouse and human tissues as well as in several viruses [2–8]. The expression levels of moRs seem to be developmentally regulated in the simple chordate, and their abundance can exceed the corresponding mature miR. Pri-miRs are transcribed from miR genes by RNA polymerase II and sequentially processed by the enzymes Drosha and Dicer into a ~20–25 nucleotide mature miR duplex [9]. Bioinformatic analysis indicated that moRs predominantly originate from the 5’- arm of the pri-miR, though some moRs derived from the 3’ arm have also been reported.Though miRs are known to play a critical role in regulating gene expression, moRs are currently considered to be simply a byproduct of miR biogenesis with no known function. Umbach et al recently demonstrated that a viral moR (moR-rR1-3-5p) has a moderate inhibitory effect on the expression of an artificial mRNA, suggesting that moR could theoretically regulate endogenous target mRNAs [7]. Previous studies demonstrated that moRs maintain high sequence conservation across species, and that miR precursors containing moRs are generally old from an evolutionary perspective [2]. All these findings together with observations that miR and moR expression are often not highly correlated [2], have led us to hypothesize that moRs may be functional transcripts. In this study, we characterized moR expression in mouse vascular smooth muscle cell (VSMC) using small RNA sequencing and showed for the first time that moR-21 alters endogenous target gene expression and is biologically active.

## Materials and Methods

### Carotid Artery Injury Model

Mice were handled in accordance with US National Institutes of Health standards, and all procedures were approved by Tufts University/Tufts Medical Center Institutional Animal Care and Use Committee (IACUC). Wild type C57/Bl6 mice were purchased from Jackson Laboratory. Mice were housed in a temperature and light-controlled colony room (12 h light/dark cycle) in groups of 4 with chow diet and water provided ad libitum. The mouse carotid injury model used in this study was performed as described previously [10]. Briefly, 19–22 gram male C57BL/6 mice were anesthetized with inhaled isoflurane (3–5% induction then 1–3% maintenance to effect via nosecone). Buprenorphine was given during the opening incision (0.05–0.1 mg/kg, SC). The left common carotid artery was denuded of its endothelium by intraluminal passage of a wire. Postoperatively, mice were housed in individual cages and given buprenorphine (0.05 mg/kg, SC, PRN) for pain control. At post injury day 3, mice were euthanized and both carotid arteries were harvested. For euthanasia, mice were deeply anesthetized with 3.5% isoflurane followed by thoracotomy and organ harvest. None of the mice died prior to carotid harvest.

### Cell lines

Mouse aortic smooth muscle cell (MAoSMC) harvest and culture: MAoSMC were obtained using the explants procedure as previously published [11]. Briefly, several mouse aortas were obtained sterilely and placed into 100mm dish containing media. The adventia was cleaned off and the aorta was cut horizontally into 10–15 pieces. Each piece was placed into a 6 or 12 well collagen Biocoat plate (Fisher). Explants were cultured in Dulbecco’s modified Eagle’s medium (DMEM) containing antibiotics and 10% bovine growth serum (BGS) for 3–7 days. When the well was 50–75% confluent, the explants were removed, and the MAoSMC were cultured in low glucose phenol red-free DMEM containing antibiotics and 10% fetal bovine serum (FBS). Before experimental use, MAoSMC were grown in low glucose DMEM supplemented with 5% dextran-coated charcoal-treated fetal bovine serum.

Human embryonic kidney 293 (HEK293), MCF-7, NIH3T3, and mouse cardiac fibroblast (MCFB) cells were cultured in DMEM medium supplemented with 10% FBS. All cells were incubated at 37°C in humidified air containing 5% CO2.

### Cell transfection

Plasmid transfection was performed with Lipofectamine LTX or Lipofectamine 2000 (Life Technologies) following the manufacturer’s protocol. Transfection with miR and moR mimetics and antisense moRs (Exiqon) was performed using RNAiMAX (Life Technologies) according to the supplier’s instructions. The final concentrations of miR/moR mimetics and antisense moRs in the Western blot experiments were 20nM and 50nM, respectively.

### Cell proliferation assay

MAoSMC were transiently transfected with miR-21, moR-21, and scrambled miR control mimetics (Life technologies) at 20nM final concentration. Cells were seeded in 96-well plates at 2000 cells/well density. Cell proliferation was measured at 0, 1, 2, 3 days post seeding using Cell TiterGlo Luminescent Cell Viability assay (Promega) following the manufacturer’s protocol. Each assay was performed in quaduplicate.

### RNA extraction and real-time quantitative PCR (RT-qPCR)

Total RNA was extracted from MAoSMC using the miRNeasy kit as described in the manufacture’s protocol (Qiagen). Concentration of the isolated RNA was quantified using a spectrophotometer. The expression levels of miR-21 and moR-21 were measured by RT-qPCR using TaqMan MicroRNA Reverse Transcription Kit and Taqman® MicroRNA Assay (Life technologies). Endogenous small nucleolar RNA 202 (snoRNA202) and U6 were used as an internal control for the amount of input RNA. Each assay was performed in duplicate. The relative expression of miR-21/moR-21 was calculated using the 2^−ΔΔCt^ method. The expression levels of target genes were measured by RT-qPCR using SuperScript III First-Strand Synthesis System (Life Technologies) and SsoFast^TM^ EvaGreen Supermix (Bio-Rad) according to manufacturer’s instructions. The primers for each target are: *Cd164*: forward GTGTTCTGTAATACCTCCTACC, reverse CTGCTGATGTGACAACTGAG; *Fnip1*: forward TCCAACCTGCTTCATTCCACTC, reverse GAACCACTCCTAGCTCCTTGAC; *Mat2a*: forward TTCTCATCCATTGTCGATCTC, reverse CCTCTGATAAATTGGCTTCTTC; *Rassf3*: forward GGCTCTGCTCAGGAAGTTTC, reverse ACTAAGGGTGTCGGTTCTGG; *Mgrn1*: forward CCTCTATTGATGATGTCCTG, reverse CTTACTCCTCTATACCAACAG; *Trp53inp1*: forward GTCACTACTTCTTCCAGCCAAG, reverse CTGAGGACTCTTCACCAATGTC; *Txndc5*: forward TGAGCCCACGGGTGACAAGG, reverse GCCACACCACGGAGCATAGAAC; *Vps54*: forward GCTCACTCGCCTGACAGATC, reverse GCAGACACCGTGAAGAGAGG; *Bmf*: forward ACAACTCGGAGGCTGAGAC, reverse TCTGACTGGAACACATCATCTTC; *Lrig1*: forward TGTTGGATACACTAGAGAGC, reverse AGTCACTACATACAGCAGAG; *Stt3b*: forward AGAGTTCCGAGTAGACAAAGC, reverse CCAATCTCAGCATTACGTGTTC; *B2M*: forward TTCTGGTGCTTGTCTCACTGA, reverse CAGTATGTTCGGCTTCCCATTC; *Gapdh*: forward GCCGGTGCTGAGTATGTCGT, reverse GGCGGAGATGATGACCCTTT. All the RT-qPCR experiments were done in biological replicates (n≥3). Duplicate technical replicates were done for each biological sample. Expression of each target gene was measured in duplicate and *B2M* or Gapdh was used as internal controls for input RNA. The relative expression of each gene was calculated using 2^−ΔΔCt^ method. RT-qPCR was performed using a Taqman 7900HT instrument (Life Technologies).

### Small RNA sequencing

Total RNA including small RNAs was extracted from cultured mAoSMC using miReasy kit (Qiagen) according to the manufacturer’s instructions. Triplicate samples were prepared. Small RNA library construction and illumina HiSeq 75bp single read sequencing were performed at the Yale Center for Genome Analysis (YCGA, http://ycga.yale.edu/). moR quantitation was performed essentially as previously described [1]. Briefly, reads were trimmed to remove adaptor sequence, and inserts longer than 13 bases were mapped to the mouse mm10 genome using Bowtie [12] (with settings–n 0 -m 5—best—strata, allowing 0 mismatches and up to 5 genomic loci with a perfect match). Reads that mapped to within 50 bp of more than one annotated mouse pri-miR (from mirBase [13]) were allotted evenly between these locations. Reads that fell entirely within established miRbase 3p or 5p miR coordinates, plus or minus 3 bp, were counted as 3p or 5p miRs, respectively. Reads that fell entirely within the range of 35 bp 3’ to 3 bp 5’ of the last base of a 3p miR were counted as 3p moR reads. Those that fell entirely between 3 bp 3’ and 35 bp 5’ of the first base of a 5p miR were counted as 5p moR reads. On average each library contained 17.4 million qualifying inserts, of which 13.8 million (79.3%) mapped to one of these four regions, indicating that the large majority of inserts represented miR or moR sequences. Reads were divided by the number of million reads mapping to these regions in each library to give normalized reads per million reads (RPMR) values. Raw and processed data is available through GEO under the accession number GSE75114.

### Microarray Expression Analysis

MAoSMC were transfected with scrambled control or moR-21 mimetics at 5nM final concentration. Triplicate samples were prepared for each treatment. Total RNA was isolated at 48hr post-transfection. Labeling and hybridization to MouseWG-6 v2.0 Expression BeadChip arrays (llumina) were performed according to the YCGA protocol. The gene expression studies were carried out using the standard protocols for MouseRef-8 v2.0 Expression BeadChip at YCGA. Briefly, the Beadstudio suite of programs were used to calculate the quantile normalized expression values for all probe sets. Bioconductor packages Lumi [14] and Limma Linear models and empirical Bayes methods [15] for assessing differential expression in microarray experiments were used to process and annotate the expression values and calculate the fold changes and *P*-values. Raw and processed data is available at GEO under accession number GSE75114.

### Plasmid constructions

The luciferase reporter pIS0 was a gift from Dr. David Bartel (Addgene plasmid #12178) [16]. To generate luciferase reporter pIS0-Txndc5, the 1.3kb sequence containing the 3’UTR of Txndc5 was generated by PCR using primers 5’- CTGAGCTCGAGACCCCGGGGAAGTCAT-3’ (SacI site underlined), 5’- CATCTAGA CATTTAAGCAAAGACCAGAC (XbaI site underlined), with mouse genomic DNA as template. The PCR fragments were cloned into pIS0 using SacI/XbaI sites. To generate pIS0-Txndc5-mut1 and mut2, overlap extension PCR was performed as previously described [17]. The flanking primers are 5’-CCCAAATACTCATGCTGTTC-3’, located in the Txndc5 3’UTR, and 5’- TCTCAAGGGCATCGGTCGAC-3’, located in pIS0 backbone. The 2 sets of internal mutagenic primers are Mut1F: 5’-TGAAAAGAAAACTCAAGTGGT**TG**AATTTGGTTTATACTTTCTAA-3’, Mut1R: 5’-TTAGAAAGTATAAACCAAATT**CA**ACCACTTGAGTTTTCTTTTCA-3’ and Mut2F: 5’-TATGAAAAGAAAACTCAAGTG**AC**ACAATTTGGTTTATACTTTCT -3’, Mut2R: 5’-AGAAAGTATAAACCAAATTGT**GT**CACTTGAGTTTTCTTTTCATA -3’. The resulting extended fragment was digested by BamHI and inserted between the two BamHI sites of pIS0-Txndc5. DNA sequencing was performed to confirm the mutagenesis.

### Design of the moR-21 sponge

The moR-21 sponge oligonucleotide and reverse complement strand were synthesized at IDT (Coralville, Iowa, USA) as a gBlocks Gene Fragment. The sequence was as follows: 5’- actagcaCTCGAGccgaTCCGACTCCTGGTACAGgcgtTCCGACGTATGGTACAGacgcTCCGACCATTGGTACAGtcgaTCCGACGTCTGGTACAGaccgTCCGACGCATGGTACAGccggTCCGACCGCTGGTACAGacgaTCCGACCTCTGGTACAGacggatcgcGGGCCCtaatatc-3’. *Xho*I and *Apa*I sites were introduced for cloning. The moR-21 sponge DNA fragment was then cloned into pCMV-d2eGFP-21 (a gift from Dr. Phil Sharp, Addgene plasmid #21972), which is a miR-21 sponge plasmid [18], using the same cloning sites (*Xho*I and *Apa*I). The control sponge plasmid, pCMV-d2eGFP-CXCR4, was a gift from Dr. Phil Sharp (Addgene plasmid #21967) [18].

### Luciferase assays

HEK293 were transiently co-transfected with the pIS0 luciferase reporters, a β-galactosidase reporter, and wild type or mutated moR-21 mimetics. 24hrs after transfection, a firefly luciferase assay was carried out as follows: 20μl cell lysate was added to 100μl of firefly luciferase assay buffer (Promega). The samples were placed in a luminometer (Luminoscan Ascent, Labsystems) and light output was determined over a 10 second interval. β-galactosidase activity was measured with a Tropix Galacto-Light Plus kit (Applied biosystems) following the manufacturer’s instruction. Firefly luciferase activity was normalized to β-galactosidase activity.

### RNA-Binding protein immunoprecipitation (RIP)-RT-PCR

RIP was performed using HEK293 cell lysate (2 X 10^7^ cell equivalents per IP), 5μL of either a normal mouse IgG (Millipore, CS200621) or Anti-Ago2 antibody (Millipore, cs204386), were used together with the Magna RIP RNA-binding protein immunoprecipitation kit, as per manufacture’s protocol (Millipore). Purified Ago2 associated RNA was subjected to qRT-PCR for detection of miR-21, moR-21 and Txndc-5.

## Results and Discussion

### moR-21 is the most abundant moR in VSMC and plays a functional role in regulating cell proliferation

**Fig 1:**
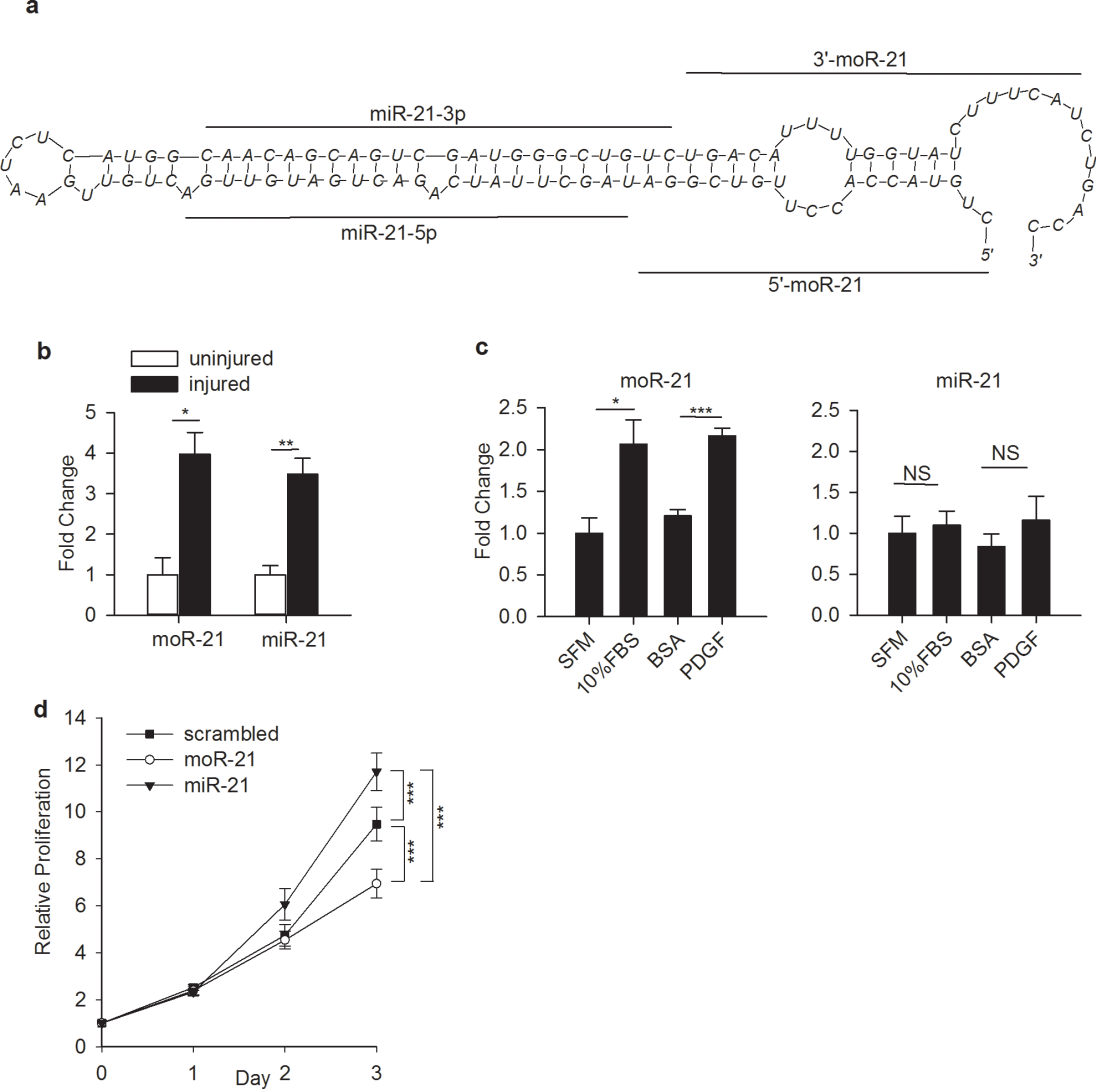
MoR-21L plays a functional role in VSMC. (a) Location of moRs and miRs sequences on the predicted secondary structure surrounding the pre-miR-21 hairpin. The RNA structure prediction software mFold was used to predict pre-miR-21 secondary structure. (b) Increased expression of moR-21 and miR-21 in injured mouse carotid artery. Data are shown as mean ± SEM and are from 3 independent experiments. (c) Abundance of moR-21 and miR-21 in VSMC cultured under different conditions. VSMC were cultured in serum free medium (SFM) and medium containing 10% fetal bovine serum (FBS) or PDGF. BSA is the control for PDGF. Data are shown as mean ± SEM and are from 4 independent experiments. (d) Effects of over-expression of moR-21, miR-21, and scrambled mimetics on VSMC proliferation. VSMC were transfected with 20nM scrambled control, moR-21, and or miR-21 mimetics. Cell proliferation was measured at 0, 1, 2, and 3 days using Cell TiterGlo. Data are presented as relative proliferation, compared with scrambled mimetic-transfected cells on day 0. Data are shown as mean ± SEM and are from 5 independent experiments. *P* values were determined by one way repeated measure ANOVA. NS: nonsignificant, *: *P*<0.05, ***: *P*<0.001.

We performed small RNA sequencing in mouse aortic smooth muscle cells (mAoSMC). We detected the expression of 34 moRs coming from 32 precursors (at an expression level of >1 read per million reads (RPMR), S1 Table). moR-21L (also called moR-21a-5p) was identified as the most abundant moR (expression level 176 RPMR). Because of its high abundance and its close connection to miR-21, known to regulate many important processes in VSMC [19–21], we chose to further study moR-21. The schematic structure of pri-miR-21 is shown in Fig 1A. As a first step to determine whether moR-21 is functional, we measured moR-21 and miR-21 abundance in wire-injured mouse carotid arteries. The relative abundance of both moR-21 and miR-21 was significantly increased in injured carotid arteries (Fig 1B). Given that the proliferation of VSMC, the major component of blood vessel walls, contributes to the pathogenesis of a wide variety of vascular diseases, we next studied the changes of moR-21 and miR-21 abundance in non-proliferative versus proliferative VSMC. We found that the expression of moR-21 was significantly increased in cells cultured in medium containing 10%FBS or platelet-derived growth factor (PDGF), a known VSMC proliferation agonist [22] (Fig 1C left panel). In contrast, no change was observed in miR-21 expression (Fig 1C right panel). These findings suggest that in different physiological states, the expression pattern of moR-21 is different from that of miR-21. We compared the levels of moR-21 and miR-21 in mAoSMC to those in three other cell lines, by qRT-PCR. We found that the U6-normalized levels of both miR-21 and moR-21 were highest in mAoSMC, relative to mouse cardiac fibroblasts (MCFBs), NIH3T3 or HEK293 cells, indicating that moR-21 and miR-21 levels vary widely between cell types (S1A & S1B Fig). We also observed that the miR-21 and moR-21 ratio varies by 20 fold between cells lines, from 64 in NIH3T3 cells to 1253 to HEK293 cells, supporting that the relative abundance of miR-21 and moR-21 also varies widely by cell type (S1C Fig). Next we measured the effect of moR-21 and miR-21 over-expression on VSMC proliferation. Though miR-21 over-expression enhances VSMC proliferation (consistent with prior publications [19,20]), strikingly, we found that moR-21 significantly decreases VSMC growth (Fig 1D). These findings indicate that moR-21 and miR-21 exert opposing effects on VSMC proliferation.

### moR-21 plays a role in gene regulation and has a different target gene set from miR-21

**Fig 2:**
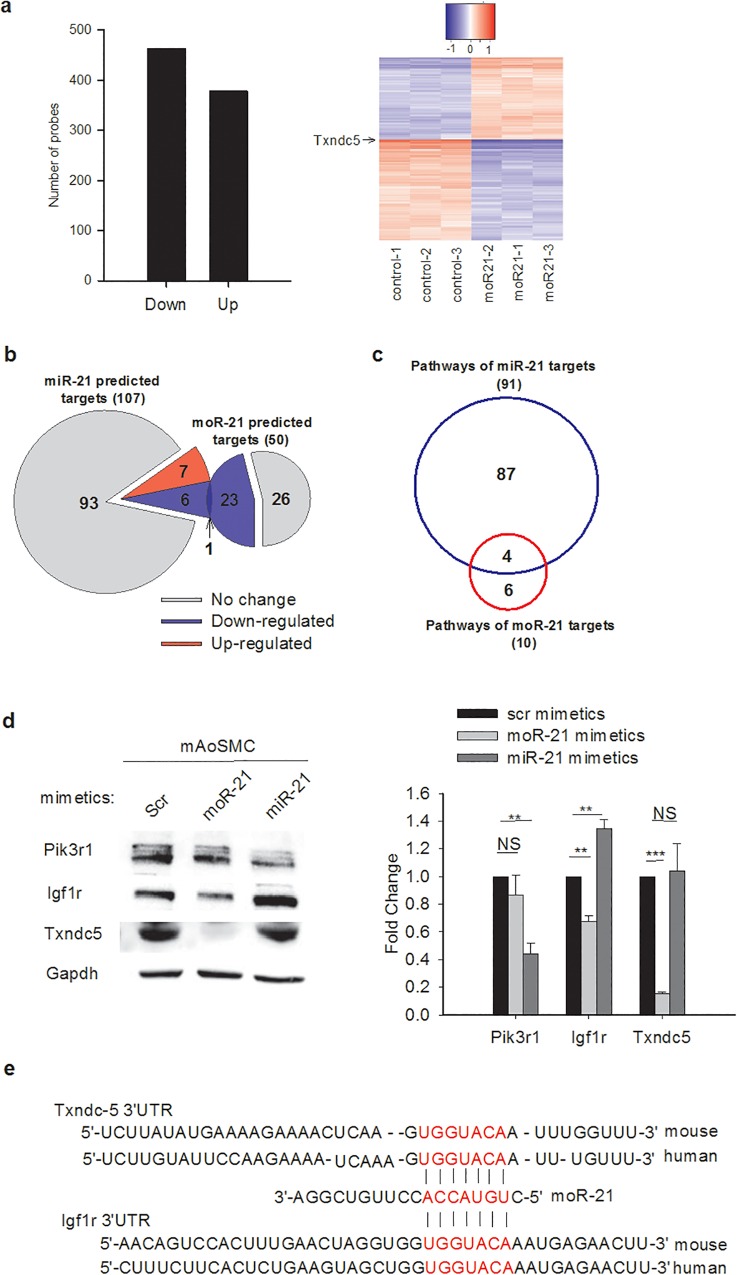
moR-21 plays a role in gene regulation and has a different target gene set from miR-21. (a) Differentially expressed transcripts in moR-21 over-expressed VSMC compared with scrambled control-treated cells. In moR-21-treated cells, 460 and 378 transcripts were down- or up-regulated, respectively. Fold change cutoff is 1.3 and adjusted *P*< 0.002. Right panel: a differential expression heatmap for all 838 genes that were significantly regulated by moR-21 showing the log base 2 expression value for each sample minus the average log base 2 expression value for all samples (three control and three moR-21 treated). (b) Comparison between miR-21 and moR-21 targets predicted by TargetScan and their regulation by moR-21, as detected by microarray in VSMC. (c) Comparison of molecular pathways that predicted miR-21 and moR-21 targets participate in. The analysis was performed using IPA with a *P*-value cutoff < 0.05. d, Pik3r1, Igf1r, and Txndc5 protein abundance in scrambled control, moR-21, or miR-21 mimetic treated mAoSMC cells. Representative western blots are presented. Densitometry analysis for western blots is shown at the bottom. e. Conserved moR-21 binding sites in the 3’UTR of Txndc-5 and Igf1r. Data are from at least 4 independent experiments and presented as fold change. Values are mean ± SEM. The significance of differences between different treatments was determined by Student’s *t*-test. NS: non-significant, **: *P*<0.01, ***: *P*<0.001.

To determine whether moR-21 plays a role in gene regulation, we performed gene profiling analysis in VSMC treated with moR-21 mimetics (synthetic small RNAs which contain the exact sequence of the endogenous miR or moR). As a negative control, cells were transfected with a random sequence miR mimetic. 838 transcripts were differentially expressed, including 460 that were down-regulated and 378 that were up-regulated (fold-change cutoff >1.3 and adjusted p-value < 0.002) (Fig 2A, S2 Table). To identify molecular pathways that these differentially expressed genes are involved in, we performed Ingenuity Pathway Analysis (IPA). Interestingly, the top molecular networks and biological functions included entries for cell survival, cell cycle, and cell proliferation, suggesting that the observed effects of moR-21 on VSMC proliferation could arise from its effects on gene regulation (S3 and S4 Tables).

For miR-mediated regulation of gene expression, complementary binding of the miR “seed” region, nucleotides 2–8 from the 5’ end of the miR, to the “seed match” region in the mRNA 3’UTR is essential for target recognition and binding [23]. To investigate whether a seed region is also important for moR-21-mediated gene regulation, we performed target gene prediction analysis for moR-21 using Targetscan 5.2 Custom (http://www.targetscan.org). We scanned mouse gene 3’UTRs for potential “seed match” sequences to nucleotides 2–8 of moR-21. 97 conserved targets with 99 conserved sites were predicted for moR-21 and 50 of these predicted targets were detected in the VSMC gene array with a signal above background noise. Among these predicted 50 targets, 24 were down-regulated in moR-21 treated cells, and 26 were unchanged in the microarray (Fig 2B). The probability of 24 or more of the 50 predicted target genes also being down-regulated by moR-21, by chance, is p. < 3e-16 (by Binomial test). To determine whether there is an overlap between predicted miR and moR-21 targets expressed in VSMC, we performed target prediction analysis for miR-21. 204 targets with 210 conserved sites were predicted for miR-21. 107 of the predicted targets were detected in VSMC with a signal above background noise, and only 7 were altered in moR-21 treated cells. Next we compared the down-regulated predicted targets of miR-21 and moR-21 and found only 1 gene was a predicted target of both moR-21 and miR-21 (Fig 2B). These results indicate that moR-21 and miR-21 have largely different target gene sets. Given the opposing effects of miR-21 and moR-21 on VSMC proliferation and the minimal overlap in predicted target genes, we hypothesized that miR-21 and moR-21 might regulate different genes that are involved in the same molecular pathway. To test this hypothesis, we performed pathway analysis for predicted moR-21 and miR-21 targets that are expressed in VSMC. IPA analysis showed that the 107 predicted targets of miR-21 participate in 91 canonical pathways, whereas the 50 predicted targets of moR-21 are involved in 10 pathways (*P* value cutoff <0.05). We further compared these pathways and found that 4 pathways are shared by miR-21 and moR-21 predicted targets (Fig 2C). The shared pathways and predicted targets are shown in S5 Table. We then studied the effects of over-expression of moR or miR-21 on genes that participate in several shared pathways. As shown in Fig 2D, for Igf1r, moR-21 significantly decreased its protein abundance, whereas miR-21 significantly increased its protein abundance, suggesting that miR and moR-21 may have opposite effects on certain genes. In contrast, the protein abundance of Pik3r1 was significantly decreased by miR-21 but not moR-21, while the protein abundance of Txndc5, one of the most highly moR-21 down-regulated genes (shown in Fig 2A heatmap), was significantly decreased by moR-21 but not miR-21, indicating that some genes are specifically regulated only by miR or moR-21. No change was observed in the protein abundance of Stat3 and Ywhaz in either miR or moR-21 mimetic treated cells (Data not shown). To determine whether moR-21 regulates the same targets in a different cell type, we measured Igf1r and Txndc5 protein abundance in moR-21 mimetic transfected MCF-7 cells. A pattern similar to that observed in VSMC, of moR-21-mediated down-regulation, was observed (S1 Fig). We examined the 3’UTR of Txndc-5 and Igf1r and found a highly conserved binding site for moR-21, which perfectly matched nucleotides 2 through 8 of moR-21 (Fig 2E).

**Fig 3:**
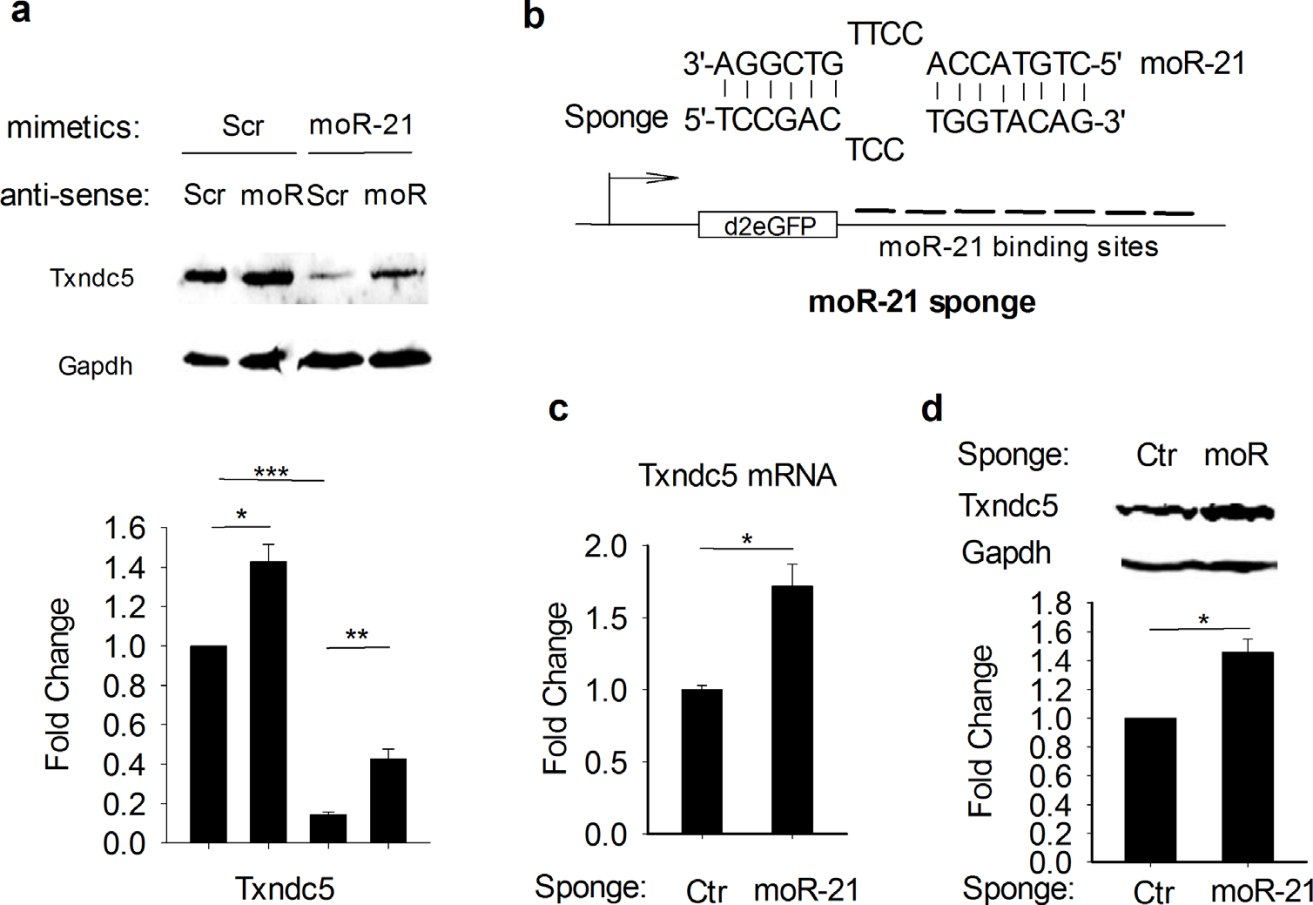
Endogenous moR-21 downregulates Txndc5 expression. (a) Txndc5 protein abundance in scrambled or moR-21 mimetic and scrambled or anti-moR-21 treated cells. Representative western blots are presented. Densitometry analysis for western blots is shown in the lower panel. Data are from at least 4 independent experiments and presented as fold change, compared with scrambled mimetic and scrambled anti-moR treated cells. (b) Upper panel: the moR-21 sponge was designed with bulged binding sites to avoid endonucleolytic cleavage by Ago2. Lower panel: the moR-21 sponge was constructed by inserting 7 copies of bulged moR-21 binding sites into the 3’UTR of a destabilized GFP reporter gene. (c) qRT-PCR analysis of Txndc5 mRNA in control or moR-21 sponge transfected HEK293 cells. Gapdh was used as an internal control for RT-qPCR. (d) immunoblot analysis of Txndc5 protein abundance in control or moR-21 sponge transfected HEK293 cells. Data are from 4 independent experiments. Values are mean ± SEM. The significance of differences between different treatments was determined by Student’s *t*-test. NS: non-significant, *: *P*<0.05, **: *P*<0.01, ***: *P*<0.001.

To confirm that the gene expression changes we observed in moR-21 mimetic over-expressing cells are not due to nonspecific or off-target effects, we examined the effect of antisense moR-21 on the abundance of Txndc5. As shown in Fig 3A, moR-21 mimetic decreased Txndc5 protein abundance, and this effect was reversed by anti-moR-21. Importantly, anti-moR-21 also increased Txndc5 protein abundance in cells transfected with scrambled moR-21 control mimetic, demonstrating reversal of the inhibitory effect of endogenous moR-21 on Txndc5 expression. To further confirm that endogenous moR-21 is functional, we performed another loss-of-function study using a ‘moR-21 sponge’. Sponges are artificial nucleotide sequences which are complementary to a miR of interest, that have been previously used as an alternative way to inhibit the function of endogenous miRs [18]. The moR-21 sponge we designed contains 7 copies of bulged moR-21 binding sites in the 3’UTR of a reporter gene (Fig 3B). As a control, we used a sponge with repeated binding sites complementary to an artificial miR (but not complementary to any known miR) ^18^. As shown in Fig 3C and 3D, both mRNA and protein abundance of Txndc5 was significantly increased in moR-21 sponge-transfected HEK293 cells (with only endogenous moR-21 expression), supporting further that the endogenous moR-21 downregulates Txndc5 expression.

### Seed region plays a key role in moR-21-mediated gene down-regulation

**Fig 4:**
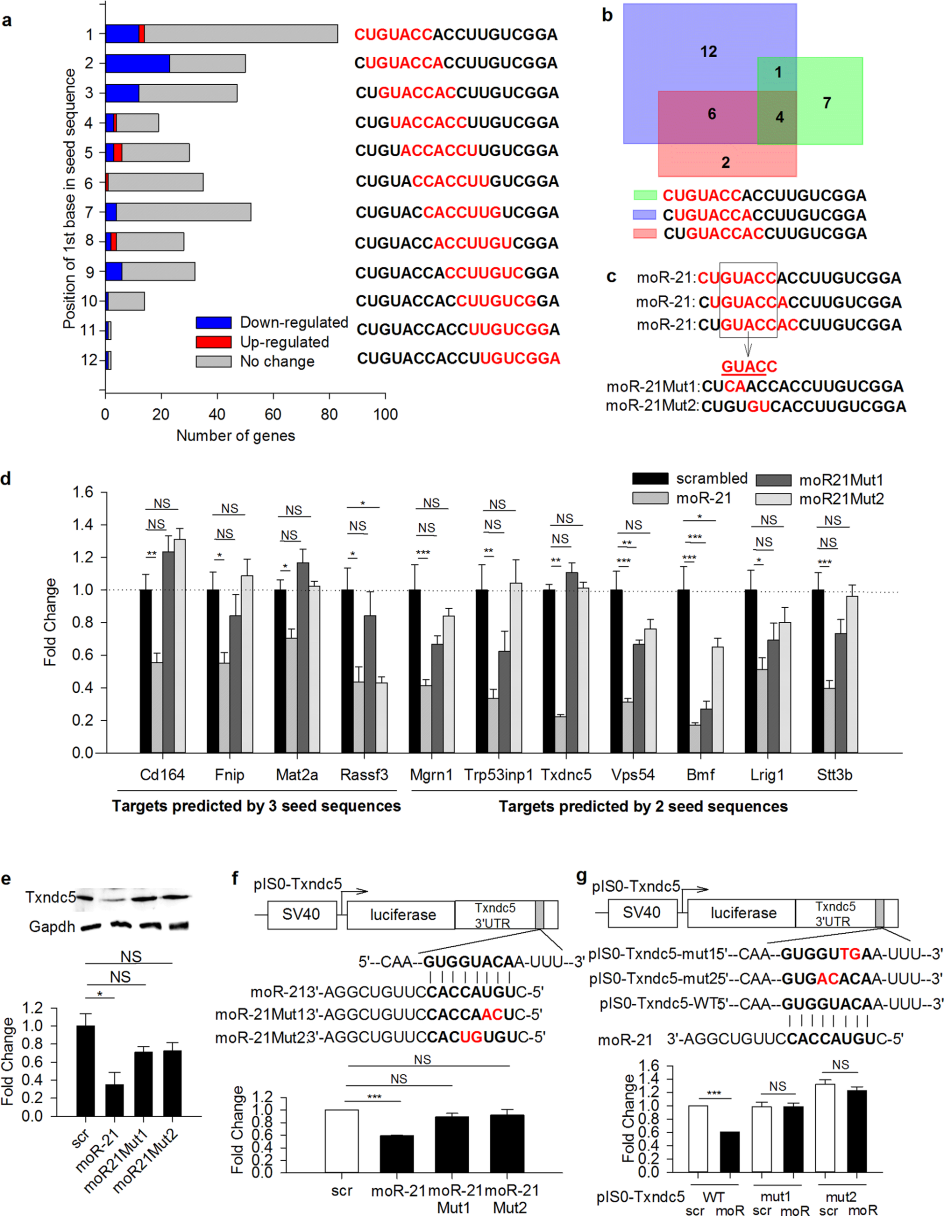
“Seed” region plays a key role in moR-21-mediated gene down-regulation. (a) Target prediction analysis for all potential “seed” sequences (highlighted in red). Numbers of predicted targets that were significantly down-regulated, up-regulated or unchanged in the microarray are represented by blue, red, and grey bars, respectively. (b) Overlap among down-regulated targets predicted by the first 3 “seed” sequences. (c) The common region of the first 3 “seed” sequences is GUACC. Each moR-21 mutant (Mut1 and Mut2) carries 2 nucleotide substitutions in the common region, which are highlighted in red. (d) Effects of wild-type and mutated moR-21 mimetics on the mRNA levels of the 11 targets genes predicted by 2 or 3 potential “seed” sequences. Each gene expression level was calculated as relative fold change compared to the scrambled control-treated sample. B2M was used as an internal control for RT-qPCR. Data are shown as mean ± SEM and are from at least 5 independent experiments. (e) Txndc5 protein abundance in scrambled control, wild type moR-21, and mutated moR-21 treated cells. Representative western blot is presented. Densitometry analysis for western blots is shown at the bottom. Data are shown as mean ± SEM and are from at least 3 independent experiments. (f) (g) Schematic presentation of Txndc5 3’UTR luciferase reporter and luciferase assay. A perfect match between the “seed” region in moR-21 and the “seed match” region in Txndc5 3’UTR is represented by short vertical lines. Nucleotide substitutions in moR-21 “seed” region (f) and Txndc5 3’UTR “seed match” region (g) are highlighted in red. Luciferase activity was normalized to beta-gal activity. Data are presented as fold change, compared with scrambled control-treated cells. Values shown are mean ± SEM of at least 3 independent experiments. The significance of differences between different treatments was determined by one-way analysis of variance (ANOVA), followed by Tukey’s test (b) or Student’s *t*-test (c), and Student’s t-test (d,e). NS: non-significant, *: *P*<0.05, **: *P*<0.01, ***: *P*<0.001.

Our initial target gene prediction analysis for moR-21 suggested that a seed region may also exist in moR-21 and play a critical role in moR-21-mediated gene regulation. Nucleotides 2–8 represent the classic seed region for miRs. To determine whether sequences other than 5’ end 2–8 might serve as a seed region for moR-21, we performed target prediction analysis for all potential seed sequences in moR-21 using Targetscan (Fig 4A). We then examined the changes of these genes in the gene profiling data. Targets predicted by nucleotides 2–8 have the highest number of regulated targets (all of them are down-regulated genes), suggesting that nucleotides 2–8 may be the most relevant seed region of moR-21. However, targets predicted for the potential seed sequences 1–7 and 3–9 also included many more down-regulated genes than targets predicted for the other potential seed sequences. To explore this observation further, we compared the down-regulated targets that were predicted by the first three seed sequences. Overlap of predicted targets amongst these three seed sequences is shown in Fig 4B. Four genes were predicted by all three seed sequences and seven more genes were predicted by two seed sequences (including Txndc5). Since these 11 genes are most likely to be direct targets of moR-21, we used these genes as candidate targets of moR-21 in the following studies. Detailed information regarding these targets is listed in the S6 Table.

To confirm the seed sequence plays a key role in moR-21-mediated gene regulation, we designed two synthetic moR-21 mutants each with two nucleotide substitutions in the common region of the first three seed sequences (Fig 4C). We then determined the effect of over-expressing the wild-type and mutated moR-21s in VSMC on the abundance of the 11 gene targets that were predicted by at least two of the first three seed sequences. MoR-21 significantly decreased the mRNA level of all 11 genes in VSMC (Fig 4D). Among the four target genes that were predicted by all three seed sequences, none were down-regulated by mutant 1 (moR21Mut1), and only one was decreased by mutant 2 (moR21Mut2) (Fig 4D). Among the seven genes that were predicted by two seed sequences, only two were regulated by moR21Mut1 and only one was regulated by moR21Mut2 (Fig 4D). These findings indicate that the seed sequence is indispensable for moR-21-mediated down-regulation of mRNA. To determine whether there are changes in abundance of the corresponding protein of the target genes, we measured protein expression of Txndc5 gene, the most down-regulated target among the 11 genes. We found wild-type, but not mutated moR-21, significantly decreases Txndc5 protein abundance (Fig 4E).

To further investigate the mechanism by which moR-21 regulates gene expression, we cloned the 3’ UTR of the Txndc5 gene into a luciferase reporter. We then measured luciferase activity in cells transfected with the luciferase reporter along with wild type or mutated moR-21 mimetics. Wild type moR-21 significantly reduced luciferase activity, whereas neither of the mutants had any significant effect (Fig 4F). These results suggest that moR-21 down-regulates Txndc5 expression by binding to its 3’UTR through its seed region. In addition, we mutated the corresponding bases in the seed match sequence located in the Txndc5 3’ UTR luciferase reporter and generated two seed match mutants (pIS0-Txndc5-mut1 and mut2) (Fig 4G). We then measured luciferase activity in the cells transfected with the wild type and mutated reporters along with moR-21. Consistent with data presented above, mutations in 3’ UTR seed match sequence abolished moR-21-mediated decrease in luciferase activity (Fig 4G). These findings support that the seed match sequence in the target 3’UTR is also indispensable for moR-21-mediated gene down-regulation.

### Ago2 is required for moR-21-mediated gene repression

**Fig 5:**
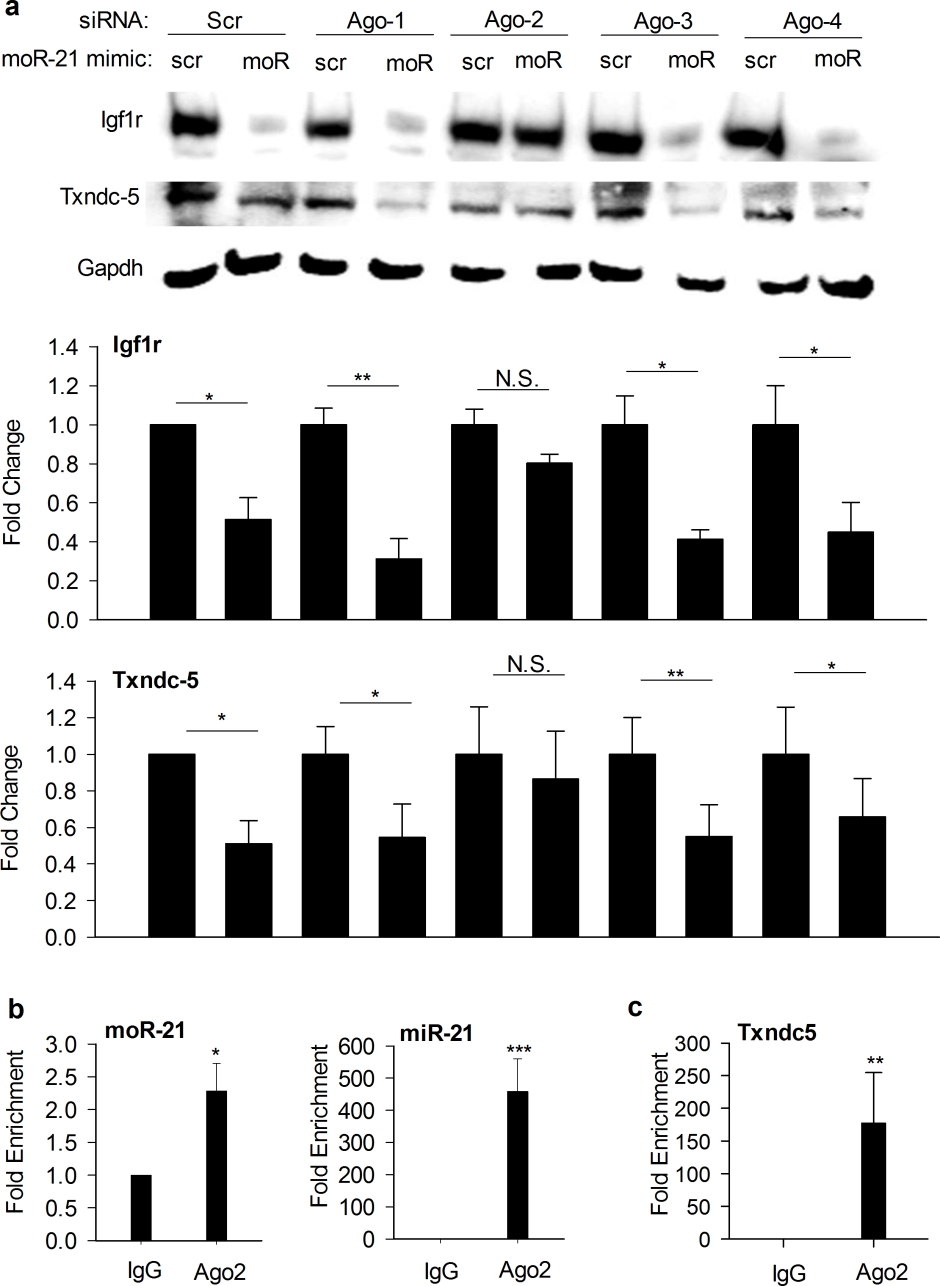
Ago2 is required for moR-21-mediated gene repression. (a) Immunoblot analysis showing the effects of knocking-down Ago1-4 on Igfr1 and Txndc5 protein abundance in moR-21 over-expressing cells. Scr: transfection control with scrambled siRNA or small RNA mimetic. Gapdh: loading control. Lower bar graphs show densitometry results in line with the representative blots. Data are from 3–5 independent experiments and presented as fold change. Values are mean ± SEM. **b,c** RIP analysis of HEK293 cell lysates to measure the interaction of Ago2 with endogenous miR-21 or moR-21 (b), and Txndc5 (c). Normal mouse IgG served as IP control. MoR-21, miR-21, or Txndc5 abundance was quantified using RT-qPCR and represented as fold enrichment in Ago2 RIP compared with IgG RIP. Data are from 4 independent experiments. Values are mean ± SEM. The significance of differences between different treatments was determined by Student’s *t*-test. NS: non-significant, *: *P*<0.05, **: *P*<0.01, ***: *P* <0.001.

After generation in the cytoplasm, mature single-stranded miRs are incorporated into the RNA-induced silencing complex (RISC) [24]. Within RISC, miRs bind to a member of the Argonaute (Ago) protein family, which direct them to the 3’UTR of target genes [25]. In mammals, the Ago family has four members, Ago1-4. All four Ago proteins interact with miRs [26,27]. Only Ago2 exhibits endoribonuclease activity and is capable of target mRNA cleavage. To determine whether Ago proteins are involved in moR-21-mediated gene repression, we studied the effects of Ago 1–4 knock-down on moR-21-mediated regulation of Igfr1 and Txndc5 in mAoSMC. As shown in Fig 5A, in Ago1, Ago3, and Ago4 siRNA transfected cells, similar to scrambled siRNA transfected cells, moR-21 decreases Igfr1 and Txndc5 protein abundance, whereas in Ago2 siRNA transfected cells, moR-21 has no effect, suggesting that Ago2 is required for moR-21-mediated gene repression. To further explore whether moR-21 associates with Ago2 *in vivo*, we performed anti-Ago2 RNA immunoprecipitation (RIP) in HEK293 cells and compared miR-21 and moR-21 abundance in IgG control RIP and Ago2 RIP using qRT-PCR. We found that both miR-21 and moR-21 were significantly enriched in Ago2 RIP compared with IgG RIP (Fig 5B), indicating that both miR-21 and moR-21 are Ago2-associated small RNAs. We then measured Txndc5 mRNA abundance in IgG RIP and Ago2 RIP and found that Txndc5 mRNA was significantly enriched in Ago2 RIP, indicating that Txndc5 mRNA is associated with Ago2 (Fig 5C).

MoRs are small RNAs that were previously known to be created from the same pri-miRs as mature miRs. However, there was no prior experimental evidence that moRs had any biological functions. Our results show, for the first time, that moR-21 plays a role in post-transcriptional gene regulation and inhibits VSMC proliferation. Our findings also indicate that miR-21 and moR-21 may regulate different genes in the same pathway and may oppose each other on regulation of certain genes, such as Igf1r. MoR-21 may regulate genes through several mechanisms. For Txndc5, moR-21 may induce mRNA degradation. For Igf1r, moR-21 may play a role in translational repression (as Igf1r mRNA was unchanged by moR-21 in the VSMC microarray study). Importantly, since miRs and moRs are derived from the same precursor and are physically located next to each other, our findings also highlight the need for reinterpretation of previous miR functional studies in which the expression of miRs was manipulated through disruption of miR loci or through over-expression of complete pri-miR sequences. In these cases, expression of moRs were also likely altered and thus the observed phenotype may have resulted from changes in both miR and moR expression. Though the current findings identify moR-21 as a previously unrecognized regulator of gene expression, they also leave many important questions unanswered. Future studies are needed to confirm whether the gene regulation mechanisms described here are also utilized by other moRs. In addition, moR-mediated gene regulation may be much more complicated than described here. In the current study, we predicted moR-21 targets by searching for “seed match” sequences only in the 3’UTR. It will be of great interest to study whether moRs could base pair with other regions of mRNA, or even noncoding RNAs, and regulate their expression. Furthermore, in the current study, although Txndc-5 was verified as a direct target of moR-21 by extensive functional studies, additional detailed molecular analyses of other potential moR-21 target genes will be important, in future studies, to further explore the functional significance of moR-21. Note also, that while the current study suggests that moR-21 promotes Txndc-5 mRNA degradation through interaction with Ago2 (in a mechanism similar to that used by miRs), future studies will be necessary to provide further details of moR-21 gene regulatory mechanisms. Our study also raises unanswered questions such as why the expression levels of moRs and miRs can be so different, and how their biogenesis and degradation are regulated. Despite these limitations and unanswered questions, taken together, the current findings reveal a new level of intricacy in the regulation of gene expression by identifying moRs as biologically active participants in the regulation of gene expression.

## Supporting Information

S1 Fig Relative expression levels of moR-21 and miR-21 in different cell types. Expression levels of moR-21 (A) and miR-21 (B) in mAoSMC, HEK293, NIH3T3, MCFB were measured by qRT-PCR and normalized to the ubiquitously expressed U6 small RNA. (C) The ratio of miR-21 expression to moR-21 expression also varied by cell type. Data are from at least 3 independent experiments. Values are mean ± SEM. The significance of differences between different treatments was determined by Student’s *t*-test. NS: non-significant, * *P*<0.05; **: *P*<0.01.
(TIF)

S2 Fig Igf1r and Txndc5 protein abundance in scrambled, moR-21, or miR-21 mimetic treated MCF-7 cells. Representative western blots are presented. Densitometry analysis for western blots is shown at the bottom. Data are from at least 4 independent experiments and presented as fold change. Values are mean ± SEM. The significance of differences between different treatments was determined by Student’s *t*-test. NS: non-significant, **: *P*<0.01, ***: *P*<0.001.
(TIF)

S1 Table Predicted moRs with genomic location and expression levels in cultured mAoSMC. (DOCX)

S2 Table Differentially expressed genes in moR-21 transfected mAoSMC. Fold change is reported directly with a fold change downwards marked with a minus sign. AveExp is the log base 2 of the normalized average expression value for all 6 samples. NC1,2,3 & moR1,2,3 are the log base 2 expression values for each of the control and moR21 treated samples. Adj. p. value is the Benjamini-Hochberg adjusted p. value.
(XLSX)

S3 Table Top 5 molecular networks predicted by IPA analysis of moR-21 targets. The p. value is determined as the chance of finding F or more of genes in the experimental gene set (which are called “focus genes”) in a network of size N, by chance, if N genes were drawn randomly from the set of all genes in their network database.
(DOCX)

S4 Table Top biological functions of moR-21 regulated genes, predicted by IPA analysis. The p. value is the probability that there would be an equal or higher overlap of the experimental gene set with the functions annotation gene set by chance, as determined by the Fisher’s exact test. The range is the range of observed p. values for overlap of the experimental gene set with each separate “functions annotation” set of genes included in the larger biological function category.
(DOCX)

S5 Table 4 common pathways in which predicted miR-21 and moR-21 targets participate. (DOCX)

S6 Table Details of targets predicted by 2 or 3 potential seed sequences of moR-21. (DOCX)

## References

[1] Shi W, Hendrix D, Levine M, Haley B. A distinct class of small RNAs arises from pre-miRNA-proximal regions in a simple chordate. Nat Struct Mol Biol. 2009;16: 183–189. 10.1038/nsmb.1536 19151725 PMC2746024

[2] Langenberger D, Bermudez-Santana C, Hertel J, Hoffmann S, Khaitovich P, Stadler PF. Evidence for human microRNA-offset RNAs in small RNA sequencing data. Bioinformatics. 2009;25: 2298–2301. 10.1093/bioinformatics/btp419 19584066

[3] Meiri E, Levy A, Benjamin H, Ben-David M, Cohen L, Dov A, et al Discovery of microRNAs and other small RNAs in solid tumors. Nucleic Acids Res. 2010; 38: 6234–6246. 10.1093/nar/gkq376 20483914 PMC2952848

[4] Bortoluzzi S, Bisognin A, Biasiolo M, Guglielmelli P, Biamonte F, Norfo R, et al Characterization and discovery of novel miRNAs and moRNAs in JAK2V617F-mutated SET2 cells. Blood. 2012;119: e120–30. 10.1182/blood-2011-07-368001 22223824

[5] Zhou H, Arcila ML, Li Z, Lee EJ, Henzler C, Liu J, et al Deep annotation of mouse iso-miR and iso-moR variation. Nucleic Acids Res. 2012;40: 5864–5875. 10.1093/nar/gks247 22434881 PMC3401436

[6] Jurak I, Kramer MF, Mellor JC, van Lint AL, Roth FP, Knipe DM, et al Numerous conserved and divergent microRNAs expressed by herpes simplex viruses 1 and 2. J Virol. 2010;84: 4659–7210. 10.1128/JVI.02725-09 20181707 PMC2863732

[7] Umbach JL, Strelow LI, Wong SW, Cullen BR. Analysis of rhesus rhadinovirus microRNAs expressed in virus-induced tumors from infected rhesus macaques. Virology. 2010;405: 592–599. 10.1016/j.virol.2010.06.036 20655562 PMC2923253

[8] Yao Y, Smith LP, Petherbridge L, Watson M, Nair V. Novel microRNAs encoded by duck enteritis virus. J Gen Virol. 2012;93: 1530–1536. 10.1099/vir.0.040634-0 22492913

[9] Bartel DP. MicroRNAs: genomics, biogenesis, mechanism, and function. Cell. 2004;116: 281–297. 14744438 10.1016/s0092-8674(04)00045-5

[10] Sullivan TR Jr, Karas RH, Aronovitz M, Faller GT, Ziar JP, Smith JJ, et al Estrogen inhibits the response-to-injury in a mouse carotid artery model. J Clin Invest. 1995;96: 2482–2488. 7593638 10.1172/JCI118307 PMC185902

[11] Karas RH, Patterson BL, Mendelsohn ME. Human vascular smooth muscle cells contain functional estrogen receptor. Circulation. 1994;89: 1943–1950. 8181116 10.1161/01.cir.89.5.1943

[12] Langmead B, Trapnell C, Pop M, Salzberg SL. Ultrafast and memory-efficient alignment of short DNA sequences to the human genome. Genome Biol. 2009;10(3): R25 10.1186/gb-2009-10-3-r25 19261174 PMC2690996

[13] Kozomara A, Griffiths-Jones S. miRBase: annotating high confidence microRNAs using deep sequencing data. Nucleic Acids Res. 2014;42(Database issue): D68–73. 10.1093/nar/gkt1181 24275495 PMC3965103

[14] Du P, Kibbe WA, Lin SM. lumi: a pipeline for processing Illumina microarray. Bioinformatics. 2008;24: 1547–1548. 10.1093/bioinformatics/btn224 18467348

[15] Smyth GK. Linear models and empirical bayes methods for assessing differential expression in microarray experiments. Stat Appl Genet Mol Biol. 2004; 3, Article3. 10.2202/1544-6115.1027 16646809

[16] Yekta S, Shih IH, Bartel DP. MicroRNA-directed cleavage of HOXB8 mRNA. Science. 2004; 304(5670): 594–596. 15105502 10.1126/science.1097434

[17] Higuchi R, Krummel B, Saiki RK. A general method of in vitro preparation and specific mutagenesis of DNA fragments: study of protein and DNA interactions. Nucleic Acids Res. 1988;16: 7351–7367. 3045756 10.1093/nar/16.15.7351 PMC338413

[18] Ebert MS, Neilson JR, Sharp PA. MicroRNA sponges: competitive inhibitors of small RNAs in mammalian cells. Nat Methods. 2007;4(9): 721–726. 17694064 10.1038/nmeth1079 PMC3857099

[19] Ji R, Cheng Y, Yue J, Yang J, Liu X, Chen H, et al MicroRNA expression signature and antisense-mediated depletion reveal an essential role of MicroRNA in vascular neointimal lesion formation. Cir Res. 2007;100: 1579–1588. 10.1161/CIRCRESAHA.106.141986 17478730

[20] Li J, Zhao L, He X, Yang T, Yang K. MiR-21 inhibits c-Ski signaling to promote the proliferation of rat vascular smooth muscle cells. Cell Signal. 2014;26: 724–729. 10.1016/j.cellsig.2013.12.013 24388835

[21] Davis BN, Hilyard AC, Lagna G, Hata A. SMAD proteins control DROSHA-mediated microRNA maturation. Nature. 2008;454: 56–61. 10.1038/nature07086 18548003 PMC2653422

[22] Bornfeldt KE, Raines EW, Nakano T, Graves LM, Krebs EG, Ross R. Insulin-like growth factor-I and platelet-derived growth factor-BB induce directed migration of human arterial smooth muscle cells via signaling pathways that are distinct from those of proliferation. J Clin Invest. 1994;93: 1266–1274. 8132765 10.1172/JCI117081 PMC294079

[23] Lewis BP, Shih IH, Jones-Rhoades MW, Bartel DP, Burge CB. Prediction of mammalian microRNA targets. Cell. 2003;115: 787–798. 14697198 10.1016/s0092-8674(03)01018-3

[24] Kim VN, Han J, Siomi MC. Biogenesis of small RNAs in animals. Nat Rev Mol Cell Biol 2009;10: 126–139. 10.1038/nrm2632 19165215

[25] Dueck A, Meister G. Assembly and function of small RNA—argonaute protein complexes. Biol Chem. 2014;395(6): 611–29. 10.1515/hsz-2014-0116 24603840

[26] Liu J, Carmell MA, Rivas FV, Marsden CG, Thomson JM, Song JJ, et al Argonaute2 is the catalytic engine of mammalian RNAi. Science. 2004;305: 1437–1441. 15284456 10.1126/science.1102513

[27] Meister G, Landthaler M, Patkaniowska A, Dorsett Y, Teng G, Tuschl T. Human Argonaute 2 mediates RNA cleavage targeted by miRNAs and siRNAs. Mol. Cell. 2004;15: 185–197. 15260970 10.1016/j.molcel.2004.07.007

