# Supplementary figures and images for: MicroRNA-Offset RNA Alters Gene Expression and Cell Proliferation
Source: PLoS One. 2016 Jun 8;11(6):e0156772. doi: 10.1371/journal.pone.0156772 (PMC4898817; doi:10.1371/journal.pone.0156772)

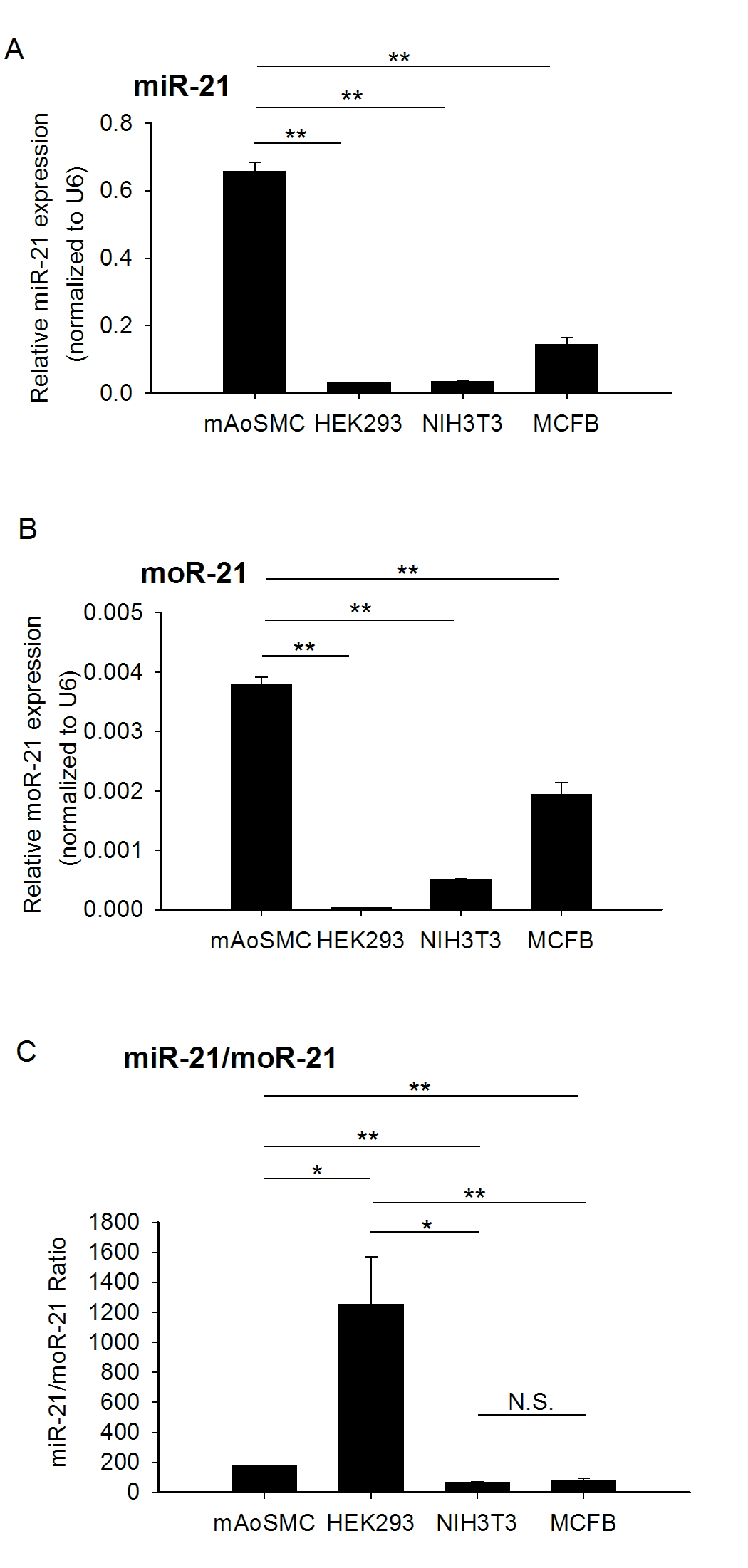

Supplement: S1 Fig — Expression levels of moR-21 (A) and miR-21 (B) in mAoSMC, HEK293, NIH3T3, MCFB were measured by qRT-PCR and normalized to the ubiquitously expressed U6 small RNA. (C) The ratio of miR-21 expression to moR-21 expression also varied by cell type. Data are from at least 3 independent experiments. Values are mean ± SEM. The significance of differences between different treatments was determined by Student’s t-test. NS: non-significant, * P<0.05; **: P<0.01. (TIF) [file pone.0156772.s001.TIF]
