# Supplementary material for: MicroRNA-Offset RNA Alters Gene Expression and Cell Proliferation
Source: PLoS One. 2016 Jun 8;11(6):e0156772. doi: 10.1371/journal.pone.0156772 (PMC4898817; doi:10.1371/journal.pone.0156772)
Supplement: S1 Table — (DOCX) [file pone.0156772.s003.docx]

**S1 Table.**

| Name | Location | Expression (RPMR) |
| --- | --- | --- |
| mmu-moR-21-5p | chr11_86584141 | 175.5 |
| mmu-moR-125a-5p | chr17_17830817 | 24.9 |
| mmu-moR-296-5p | chr2_174267113 | 22.7 |
| mmu-moR-3074-1-3p | chr13_63301211 | 21.7 |
| mmu-moR-24-1-5p | chr13_63301213 | 21.7 |
| mmu-moR-125b-5p | chr16_77646279 | 14.8 |
| mmu-moR-3102-3p.2-3p | chr7_100882330 | 14.4 |
| mmu-moR-328-5p | chr8_105308440 | 9.3 |
| mmu-moR-21a-3p | chr11_86584082 | 5.6 |
| mmu-moR-125b-5p | chr9_41581940 | 4.1 |
| mmu-let-7f-moR-5p | chrX_151912353 | 4.0 |
| mmu-let-7b-moR-5p | chr15_85707325 | 3.9 |
| mmu-let-7a-2-moR-5p | chr9_41536732 | 3.8 |
| mmu-moR-503-5p | chrX_53054049 | 3.4 |
| mmu-moR-30c-5p | chr4_120769606 | 3.3 |
| mmu-moR-324-5p | chr11_70012060 | 2.5 |
| mmu-let-7a-1-moR-5p | chr13_48538260 | 2.3 |
| mmu-moR-421-5p | chrX_103572986 | 2.1 |
| mmu-moR-33-5p | chr15_82198127 | 2.1 |
| mmu-moR-15b-5p | chr3_69009775 | 2.1 |
| mmu-moR-31-5p | chr4_88910635 | 2.0 |
| mmu-moR-23a-5p | chr8_84208527 | 1.7 |
| mmu-moR-145a-5p | chr18_61647888 | 1.6 |
| mmu-moR-125b-1-3p | chr9_41582001 | 1.6 |
| mmu-moR-542-5p | chrX_53049474 | 1.5 |
| mmu-moR-16-2-5p | chr3_69009918 | 1.5 |
| mmu-moR-16-1-5p | chr14_61631957 | 1.4 |
| mmu-moR-26a-5p | chr10_126995543 | 1.4 |
| mmu-moR-674-5p | chr2_117185151 | 1.3 |
| mmu-moR-214-5p | chr1_162223397 | 1.3 |
| mmu-moR-322-5p | chrX_53054327 | 1.2 |
| mmu-moR-130a-3p | chr2_84741116 | 1.1 |
| mmu-moR-574-5p | chr5_64970328 | 1.1 |
| mmu-moR-27a-5p | chr8_84208685 | 1.0 |
| mmu-moR-30a-5p | chr1_23272274 | 1.0 |
