# Supplementary material for: MicroRNA-Offset RNA Alters Gene Expression and Cell Proliferation
Source: PLoS One. 2016 Jun 8;11(6):e0156772. doi: 10.1371/journal.pone.0156772 (PMC4898817; doi:10.1371/journal.pone.0156772)
Supplement: S3 Table — The p. value is determined as the chance of finding F or more of genes in the experimental gene set (which are called “focus genes”) in a network of size N, by chance, if N genes were drawn randomly from the set of all genes in their network database. (DOCX) [file pone.0156772.s005.docx]

**S3 Table.**

| Associated Network Functions | P value |
| --- | --- |
| Cell Death and Survival, Cell cycle, Cellular Development | 1E-49 |
| Cellular Compromise, Cellular Function and Maintenance, Cellular Growth and Proliferation | 1E-41 |
| Hereditary Disorder, Neurological Disease, Skeletal and Muscular Disorders | 1E-34 |
| Cell Cycle, Hair and Skin Development and Function, Cardiovascular Disease | 1E-30 |
| Cell-to-Cell Signaling and interaction, Developmental Disorder, Ophthalmic Disease | 1E-30 |
