# Supplementary material for: MicroRNA-Offset RNA Alters Gene Expression and Cell Proliferation
Source: PLoS One. 2016 Jun 8;11(6):e0156772. doi: 10.1371/journal.pone.0156772 (PMC4898817; doi:10.1371/journal.pone.0156772)
Supplement: S4 Table — The p. value is the probability that there would be an equal or higher overlap of the experimental gene set with the functions annotation gene set by chance, as determined by the Fisher’s exact test. The range is the range of observed p. values for overlap of the experimental gene set with each separate “functions annotation” set of genes included in the larger biological function category. (DOCX) [file pone.0156772.s006.docx]

**S4 Table.**

| Bio Functions | Name | # Molecules | P value |
| --- | --- | --- | --- |
| Diseases and Disorders | Cancer | 334 | 6.84E-10 – 8.85E-03 |
|  | Gastrointestinal Disease | 138 | 6.84E-10 - 7.32E-03 |
|  | Dermatological Disease and Conditions | 88 | 1.66E-09 – 5.89E-03 |
|  | Neurological Disease | 175 | 4.64E-09 – 7.51E-03 |
|  | Skeletal and Muscular Disorders | 150 | 4.64E-09 – 8.14E-03 |
| Molecular and Cellular Functions | Cellular Growth and Proliferation | 216 | 7.44E-08 – 8.33E-03 |
|  | Cell Death and Survival | 215 | 3.15E-07 – 8.97E-03 |
|  | Cellular Movement | 133 | 4.82E-07 – 8.84E-03 |
|  | Cell Cycle | 90 | 9.40E-07 – 8.97E-03 |
|  | Cellular Assembly and Organization | 117 | 1.86E-06 - 8.84E-03 |
| Physiological System Development and Function | Cardiovascular System Development and Function | 111 | 3.16E-07 – 8.97E-03 |
|  | Organismal Development | 140 | 3.16E-07 – 8.97E-03 |
|  | Organismal Survival | 166 | 2.81E-06 - 2.81E-06 |
|  | Connective Tissue Development and Function | 71 | 9.79E-05 - 7.79E-03 |
|  | Tissue Development | 79 | 1.85E-04 – 8.97E-03 |
