# Supplementary material for: MicroRNA-Offset RNA Alters Gene Expression and Cell Proliferation
Source: PLoS One. 2016 Jun 8;11(6):e0156772. doi: 10.1371/journal.pone.0156772 (PMC4898817; doi:10.1371/journal.pone.0156772)
Supplement: S5 Table — (DOCX) [file pone.0156772.s007.docx]

**S5 Table.**

| Canonical Pathway | Predicted miR-21 targets | Predicted moR-21 targets |
| --- | --- | --- |
| Myc Mediated Apoptosis Signaling | PIK3R1,BCL2 | IGF1R,YWHAZ |
| IGF-1 Signaling | PIK3R1,STAT3,RASA1 | IGF1R,YWHAZ |
| Role of NFAT in Cardiac Hypertrophy | TGFBR2,PIK3R1,MAP3K1,MAP2K3 | IGF1R,SLC8A1 |
| AMPK Signaling | PIK3R1,MAP2K3,PFKM | KAT2B,SMARCA2 |
