# Supplementary material for: MicroRNA-Offset RNA Alters Gene Expression and Cell Proliferation
Source: PLoS One. 2016 Jun 8;11(6):e0156772. doi: 10.1371/journal.pone.0156772 (PMC4898817; doi:10.1371/journal.pone.0156772)
Supplement: S6 Table — (DOCX) [file pone.0156772.s008.docx]

**S6 Table.**

| Gene Symbol | Gene Name | Fold Change in microarray |
| --- | --- | --- |
| Cd164 | CD164 antigen | -2.4 |
| Fnip1 | Folliculin interacting protein 1 | -1.36 |
| Mat2a | Methionine adenosyltransferase II, alpha | -1.34 |
| Rassf3 | Ras association (RalGDS/AF-6) domain family member 3 | -1.44 |
| Mgrn1 | Mahogunin, ring finger 1 | -2.13 |
| Trp53inp1 | Transformation related protein 53 inducible nuclear protein 1 | -1.32 |
| Txndc5 | Thioredoxin domain containing 5 | -4.05 |
| Vps54 | Vacuolar protein sorting 54 (yeast) | -1.63 |
| Bmf | Bcl2 modifying factor | -1.70 |
| Lrig1 | Leucine-rich repeats and immunoglobulin-like domains 1 | -1.63 |
| Stt3b | STT3, subunit of the oligosaccharyltransferase complex, homolog B (S. cerevisiae) | -2.77 |
